# Kelp forests collapse reduces understorey seaweed β-diversity

**DOI:** 10.1093/aob/mcad154

**Published:** 2023-10-10

**Authors:** Cristina Piñeiro-Corbeira, Sara Barrientos, Isabella Provera, Manuel E García, Pilar Díaz-Tapia, Viviana Peña, Ignacio Bárbara, Rodolfo Barreiro

**Affiliations:** BioCost Research Group, Facultad de Ciencias, and CICA – Centro Interdisciplinar de Química e Bioloxía, Universidad de A Coruña, A Coruña, Spain; BioCost Research Group, Facultad de Ciencias, and CICA – Centro Interdisciplinar de Química e Bioloxía, Universidad de A Coruña, A Coruña, Spain; Department of Integrative Marine Ecology, Stazione Zoologica Anton Dohrn, 80121, Naples, Italy; Department of Ecology and Marine Resources, Instituto de Investigaciones Marinas (IIM-CSIC), Eduardo Cabello 6, 36208 Vigo, Spain; BioCost Research Group, Facultad de Ciencias, and CICA – Centro Interdisciplinar de Química e Bioloxía, Universidad de A Coruña, A Coruña, Spain; Instituto Español de Oceanografía (IEO-CSIC), Centro Oceanográfico de A Coruña, Paseo Marítimo Alcalde Francisco Vázquez, 10, 15001, Coruña, Spain; BioCost Research Group, Facultad de Ciencias, and CICA – Centro Interdisciplinar de Química e Bioloxía, Universidad de A Coruña, A Coruña, Spain; BioCost Research Group, Facultad de Ciencias, and CICA – Centro Interdisciplinar de Química e Bioloxía, Universidad de A Coruña, A Coruña, Spain; BioCost Research Group, Facultad de Ciencias, and CICA – Centro Interdisciplinar de Química e Bioloxía, Universidad de A Coruña, A Coruña, Spain

**Keywords:** *Laminaria ochroleuca*, β-diversity, kelp forest, turf-forming species, understorey

## Abstract

**Background and Aims:**

Kelps are the primary foundation species in temperate subtidal rocky shores worldwide. However, global change is causing their decline with consequences for the organisms that rely on them. An accurate assessment of these consequences may depend on which attributes of the associated community are considered. This study shows that conventional α-diversity approaches may overlook some of these consequences compared to spatially explicit approaches such as with β-diversity.

**Methods:**

A 1-year seasonal study was conducted to compare the macroalgal understorey between healthy reefs with a *Laminaria ochroleuca* canopy and degraded reefs where the canopy collapsed years ago due to excessive fish herbivory. At each reef, the understorey seaweed assemblage was recorded in five replicate quadrats to estimate α-diversity (total richness, species density, Shannon index) and β-diversity (intra- and inter-reef scale).

**Key Results:**

The understorey assemblage exhibited a distinct seasonal dynamic in both healthy and degraded reefs. α-Diversity attributes increased in spring and summer; turf-forming algae were particularly dominant in degraded reefs during summer. β-Diversity also showed seasonal variability, but mostly due to the changes in degraded reefs. None of the α-diversity estimates differed significantly between healthy and degraded reefs. In contrast, spatial β-diversity was significantly lower in degraded reefs.

**Conclusions:**

Although the loss of the kelp canopy affected the composition of the macroalgal understorey, none of the conventional indicators of α-diversity detected significant differences between healthy and degraded reefs. In contrast, small-scale spatial β-diversity decreased significantly as a result of deforestation, suggesting that the loss of kelp canopy may not significantly affect the number of species but still have an effect on their spatial arrangement. Our results suggest that small-scale β-diversity may be a good proxy for a more comprehensive assessment of the consequences of kelp forest decline.

## INTRODUCTION

Healthy kelp forests are structurally complex habitats characterized by a multi-layered understorey of algae aggregations with diverse canopy adaptations, similar in appearance to terrestrial forests (Foster, 1975; Dayton, 1985). As the main foundation species of temperate rocky shores worldwide, kelps play a key role in supporting biodiversity by creating locally stable environmental conditions that provide habitat and food for many flora and fauna (Lamy *et al.*, 2020; Smale *et al.*, 2020). Indeed, the ecological and economic importance of kelp forests in temperate latitudes rivals that of coral reefs in tropical waters (Steneck and Johnson, 2014). In addition, it has been recently suggested that kelp forests may have far-reaching beneficial effects because a significant portion of their primary production could be exported as detritus to neighbouring and distant habitats, including the deep sea, contributing to the long-term storage of blue carbon (Krause-Jensen and Duarte, 2016; Krumhansl *et al.*, 2016).

Like many other ecosystems, kelp forests have been negatively affected by climate change, with declines detected in up to 38 % of ecoregions in recent decades (Krumhansl *et al.*, 2016). Nonetheless, their response to global change differs from that of other foundation species, as they appear to be strongly influenced by local stressors and regional variability (Krumhansl *et al.*, 2016). Moreover, since at least the 1990s, the decline of kelps and other canopy-forming algae has often been accompanied by large-scale replacement by turf algae on many temperate coasts around the world, particularly near the edges of their distribution or in areas under human pressure (Filbee-Dexter *et al.*, 2016; Wernberg *et al.*, 2016; Feehan *et al.*, 2019). Evidence suggests that the replacement of kelp forests by turfs may be caused by the cumulative effects of multiple stressors (e.g. warming, eutrophication, species invasions, pollution), extreme events (e.g. marine heat waves) or even disrupted biotic interactions (e.g. herbivory, epiphytism), often indirectly driven by increased temperatures (Strain *et al.*, 2014; Filbee-Dexter and Wernberg, 2018; United Nations Environment Programme, 2023). This phenomenon, sometimes referred to as community-wide tropicalization when the decline is accompanied by the arrival of warm-water species (Wernberg *et al.*, 2016; Vergés *et al.*, 2019), has been argued to represent a persistent regime shift stabilized by feedback mechanisms (Wernberg *et al.*, 2016; Filbee-Dexter and Wernberg, 2018; Feehan *et al.*, 2019).

Macroalgal deforestation has been shown to have significant impacts on coastal ecosystems, including reduced biomass and loss of ecosystem services (Vergés *et al.*, 2014; Peleg *et al.*, 2020). Similarly, from a biodiversity perspective, communities associated with kelp forests, including fishes, invertebrates and understorey algae, can be highly responsive to canopy disturbance, and even partial canopy loss can result in rapid compositional changes within a few months (Ling, 2008; Witman and Lamb, 2018; Wernberg *et al.*, 2020). However, predicting the trajectory of these community changes following canopy disturbance can be challenging, as they depend on the dispersal and colonization traits of the local species pool (Wernberg *et al.*, 2020). Furthermore, despite evidence that variability at the reef or quadrat scale can contribute significantly to the overall diversity of kelp forests (Smale *et al.*, 2011), the question of whether small-scale spatial variability can be affected by kelp deforestation has received little attention (Piazzi and Ceccherelli, 2020). In this regard, recent research investigating the consequences of forest-to-turf replacement on seascape habitat structure shows that although turfs can harbour considerable biodiversity, they are more structurally homogeneous across regions than marine forests (Pessarrodona *et al.*, 2021). However, whether structural homogeneity may also result in a more spatially uniform assemblage (i.e. lower β-diversity) is still poorly understood. On a larger scale, biotic homogenization due to anthropogenic reduction of environmental variability, invasive species and range shifts driven by global change has been observed in many terrestrial communities (e.g. Karp *et al.*, 2012; Rodrigues *et al.*, 2013; Gámez-Virués *et al.*, 2015; Gossner *et al.*, 2016), and there is growing concern that it may affect ecosystem functioning (Mori *et al.*, 2018). Examples of biotic homogenization have been less frequently reported in the marine environment (Magurran *et al.*, 2015). Nevertheless, the biotic tropicalization and structural simplification of key temperate marine ecosystems, such as kelp forests, undoubtedly represent a form of homogenization (Agostini *et al.*, 2021; Pessarrodona *et al.*, 2021; Smith *et al.*, 2021).

It has been suggested that accurately capturing the full diversity picture requires the use of multiple response variables, rather than relying on simple metrics such as species richness in isolation (O’Shaughnessy *et al.*, 2023). This approach seems particularly relevant when considering the effects of human-induced disturbances that may have an impact on habitat structure, in addition to species richness. For example, global change, combined with local drivers, is causing a shift from temperate marine forests to turfs, which have a simpler and flattened structure but may still contain considerable species diversity (Filbee-Dexter and Wernberg, 2018; Pessarrodona *et al.*, 2021). In these and other cases, including metrics that incorporate a spatial component may be essential for an accurate assessment of the magnitude of the impact. β-Diversity, generally defined as the variation in the identities of species among sampling units, is a conventional metric in community ecology that considers the spatial component (Whittaker, 1960, 1972; Anderson *et al.*, 2011). Although is not as common a metric in marine ecology as α-diversity (Piazzi and Ceccherelli, 2020), β-diversity has been used to understand the effects of various human perturbations on the structure of biological communities at multiple scales (e.g. Chou and Lim, 1986; Moschella *et al.*, 2005; Occhipinti-Ambrogi and Galil, 2010; Bevilacqua *et al.*, 2012; García-Tejero *et al.*, 2018; Lai *et al.*, 2018; Morri *et al.*, 2019). The different measures and meanings of β-diversity have long been a source of some controversy and confusion (Anderson *et al.*, 2011). Two concepts of β-diversity are commonly distinguished: turnover and variation. The former identifies the directional change in community structure along a gradient while the latter corresponds to the non-directional heterogeneity found within a given spatial, temporal or environmental extent (Whittaker, 1960, 1972). Similarly, measures of β-diversity are often separated into classical metrics, derived directly from regional and local measures of diversity, and multivariate measures, based on pairwise resemblances among sample units (Anderson *et al.*, 2011).

In the Northeast Atlantic, several studies have documented the decline of kelp forests over the last decade (Yesson *et al.*, 2015; Araújo *et al.*, 2016; Piñeiro-Corbeira *et al.*, 2016; Barrientos *et al.*, 2020). The proposed drivers of these losses are rising temperatures (Piñeiro-Corbeira *et al.*, 2016; Barrientos *et al.*, 2020), sea urchin grazing (Fredriksen *et al.*, 2020) and, more recently, fish herbivory (Barrientos *et al.*, 2022*a*, *b*). Despite the growing body of knowledge on kelp forest shifts in this region, limited information still exists on ecosystem reconfiguration following the disappearance of these forests. Previous studies have focused primarily on the proliferation of warm-water, invasive and turf-forming species, with fewer investigations addressing the consequences of kelp forest replacement by a turf-dominated seascape (Díez *et al.*, 2012; Muguerza *et al.*, 2017). In this study, we employed overgrazed kelp reefs in NW Spain, which were previously dominated by the golden kelp *Laminaria ochroleuca*, as a model system to evaluate the utility of α- and β-diversity metrics in discerning the repercussions of this disturbance on the biotic community associated with these key coastal marine ecosystems. Given the spatial biotic homogenization that typically results from kelp loss, we hypothesized a detrimental influence on the local-scale β-diversity of the macroalgal understorey, while not necessarily an impact on α-diversity. Like other temperate ecosystems, the richness of the understorey assemblage varies with seasons. Hence, our sampling plan included a seasonal framework in which the comparison between healthy and degraded reefs was repeated seasonally over a year to ascertain if these seasonal changes affected the observed patterns.

## MATERIAL AND METHODS

### Sampling reefs

**Fig. 1.**
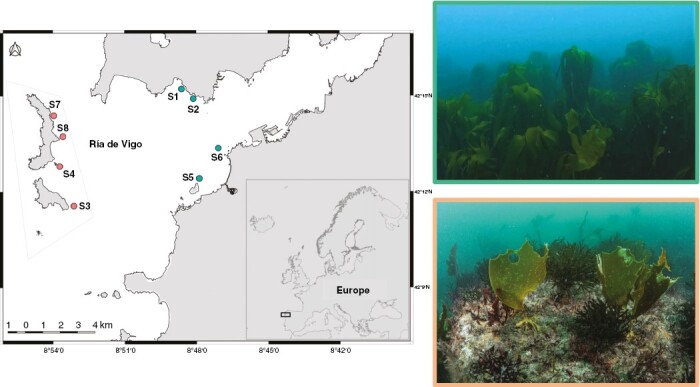
Map of the study area with the location of the studied reefs and examples of a healthy (green) and disturbed kelp forest (red).

In 2020, seasonal surveys (spring, summer, autumn and winter) were conducted at eight subtidal rocky reefs located in Ría de Vigo (NW Spain). Four reefs exhibited a perennial canopy of the perennial brown kelp *L. ochroleuca* (hereafter, healthy reefs) while the remaining four had experienced the loss of the canopy-forming kelp several years previously (degraded reefs). In these degraded reefs, only recruits and young kelps are temporarily present during the spring and summer, but they are subject to complete overgrazing each autumn (but see Barrientos *et al.*, 2022*a* for more information) (Fig. 1). Reefs were selected based on previous knowledge of the study area, ensuring that all reefs shared similar environmental conditions, including depth (5–8 m), wave exposure (semi-exposed, see Supplementary Data SI1), substrate (rocky or rocky with sand) and temperature (14–18 °C). Water temperature and dissolved inorganic nutrients (nitrate and phosphate) are strongly influenced by a seasonal coastal upwelling that brings nutrient-laden, cool deep water to the coast in spring–summer months. As a result, water temperature is largely stable in winter–autumn but fluctuates in spring–summer with the upwelling pulses. On the other hand, dissolved nutrients peak in winter, decreasing in spring and summer when the primary productivity of the area increases thanks to the subsidy provided by the upwelling (see Barrientos *et al.*, 2022*a* for further details).

### Sampling approach

During each seasonal survey, five replicate quadrats (0.25 m^2^) were haphazardly sampled within each of the eight kelp reefs. Quadrats were positioned at least 3 m apart and placed centrally within the kelp stand. All samples were collected exclusively from mono-specific forests of *L. ochroleuca*. In each quadrat, the percentage cover of seaweeds was visually estimated. Species identification was carried out to the lowest possible taxonomic level. In cases where field identification was not feasible, samples were taken to the laboratory for accurate taxonomic identification. All species were classified into four vegetation layer categories (canopy, sub-canopy, turf and crust) according to their morphological traits using the authors’ expert knowledge.

### Data analysis

The cover data from sampling quadrats were utilized to estimate total cover, and α- and β diversity of the understorey seaweed assemblage. α-Diversity was assessed as (1) species richness, expressed as both the total number of species recorded at each sampling reef and as species density (mean number of species recorded per sampling quadrat), and (2) Shannon index (Shannon, 1948). β-Diversity variation was determined as the average inter-sample dissimilarity within a given spatial extent (Whittaker, 1960, 1972) based on pairwise Jaccard dissimilarities (Jaccard, 1912) calculated using the betapart 1.5.4 package (Baselga *et al.*, 2023) in R (R Core Team, 2021). Jaccard distances are commonly used in β-diversity estimates, and they are interpretable as the probability that two species drawn at random from each sample will not be shared (Anderson *et al.*, 2006). Furthermore, multivariate estimates based on Jaccard distances are expected to give results similar to those obtained from classic metrics such as Whittaker’s β_W_ (Anderson *et al.*, 2011). β-Diversity was inferred at two spatial scales. Intra-reef β-diversity (tens of metres) was calculated by computing Jaccard distances between quadrats sampled at the same reef. On the other hand, inter-reef β-diversity was based on Jaccard distances calculated between quadrats sampled at pairs of reefs with similar health status (Supplementary Data Table S1). Given that the healthy reefs group included sites located on opposite shores of the estuary, inter-reef estimates were restricted to pairs of adjacent sites rather than including all possible pairwise comparisons between reefs in each group. This prevented the inclusion of pairwise comparisons between reefs on opposite shores from artificially inflating the estimates of inter-reef β-diversity for the healthy reefs and kept the spatial distance for inter-reef estimates similar in all cases (hundreds to a few thousand metres). To examine the influence of kelp forest health status on richness and diversity estimates, generalized linear mixed models (GLMMs) were fitted with kelp forest status (categorical, two levels) and sampling season (categorical, four levels) as crossed fixed factors and, when applicable (species density, diversity), sampling reef or pair of sampling reefs (for inter-reef β-diversity only) as a random effect. Model fitting and selection were performed using the glmmTMB package (Brooks *et al.*, 2017). For each response variable, various error distributions and link functions were tested, and model comparison was conducted using the Akaike Information Criteria (AIC) implemented in bbmle (Bolker and R Development Core Team, 2022). Model validation was carried out with the simulation-based approach of DHARMa, which provides residual diagnostics to detect typical model misspecification issues (Hartig, 2022). The significance of the fixed factors was determined using Wald χ^2^ statistics.

## RESULTS

### Composition of seaweed assemblage

**Fig. 2.**
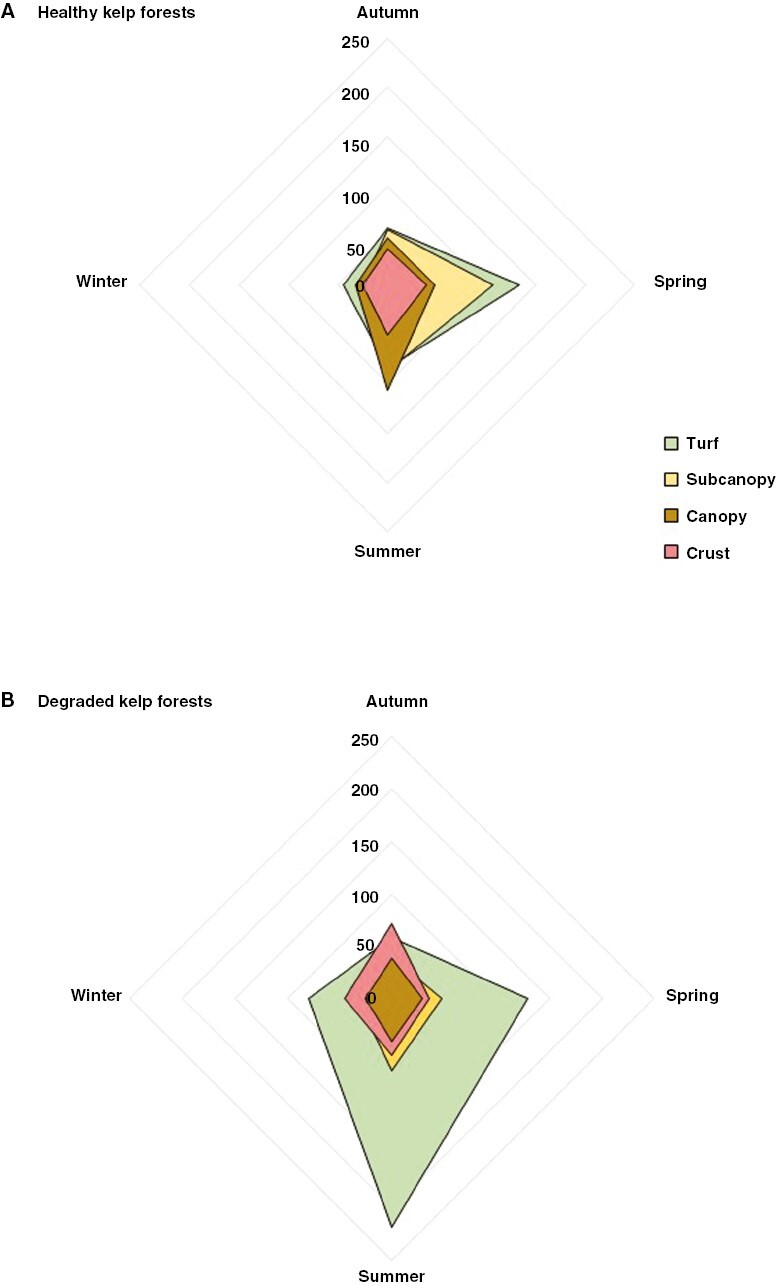
Vegetation layer abundance on (A) healthy reefs and (B) degraded reefs across seasons. The higher the area covered in the spider plot, the higher the abundance of each vegetation layer.

**Fig. 3.**
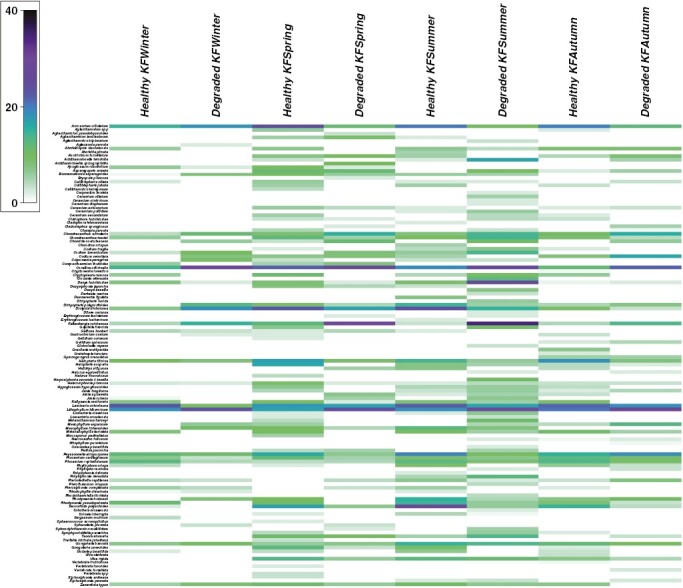
Shade plot for the abundance of each species, health status level and season represented by the shade of blue and green, from white (absent) to dark blue (most abundant). The colour shading scale within individual cells is linearly proportional to square-root-transformed abundances.

A total of 123 seaweed – nine Chlorophyta, 21 Ochrophyta and 93 Rhodophyta – species were identified in our reefs over the study period (Supplementary Data Table S2). Most of them belonged to turf (66) and subcanopy (39) vegetation layers, while 12 were ranked as canopy and six as crusts. However, species richness changed across seasons with only 31 seaweeds found in all seasons, indicating that a considerable portion of the recorded species were rare, seasonal and/or ephemeral (e.g. *Dictyota dichotoma* or *Dasya hutchinsiae*). Thus, 12 were only recorded in spring, 24 in summer, nine in autumn and just two in winter. The total cover of species from the different vegetation layers was very similar on healthy reefs, with an increase of turf and subcanopy species in summer, while on degraded reefs algal crust algae were the dominant taxa, followed by turf in spring and summer (Fig. 2). Overall, *Lithophyllum hibernicum* and *Corallina officinalis* dominated year-round on degraded reefs, except in spring and summer when *Asparagopsis armata* stadium *Falkenbergia rufolanosa* was the dominant morphotype, and to a lesser extent *L. ochroleuca*. On healthy reefs, *L. ochroleuca* was the dominant canopy-forming species except during warmer seasons, when *Acrosorium ciliolatum*, *Dictyota dichotoma* (spring) and *Saccorhiza polyschides* (summer) were more abundant (Fig. 3).

### Richness and diversity estimates

**Fig. 4.**
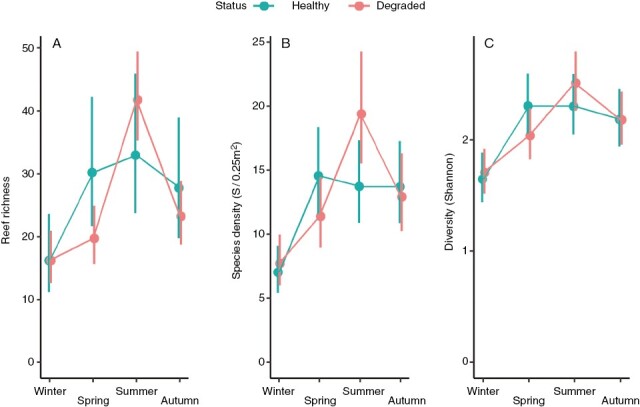
α-Biodiversity of the associated seaweed assemblage: influence of the health status (healthy vs. degraded) of golden kelp reefs on (A) total number of seaweed species per reef, (B) species density and (C) Shannon diversity. Values are least-squares means (±95 % confidence intervals) derived from GLMMs in Table 1. *N* = 4 for means in A (four reefs per level of health status) and 20 for means in B and C (four reefs per level of health status, five sampling quadrats per reef).

Total species richness in the studied area gradually increased from 44 in winter to 83 in spring and 94 in summer, before falling back to 65 in autumn. Although this seasonal trend occurred on both healthy and degraded kelp forests, the total number of species rapidly peaked from 34 in winter to 70 in spring in healthy reefs, while a gentler increase occurred in degraded reefs as total richness rose from 31 in winter to 48 in spring and to 75 in summer. This seasonal pattern was equally applicable to the mean number of species per reef. Again, mean richness per reef peaked in summer on both healthy (33.0 ± 5.56 species per reef, least-squares mean ± s.e.) and degraded reefs (41.7 ± 3.59 species per reef), and the spring surge was more pronounced on healthy than on degraded reefs (30.3 ± 5.16 and 19.7 ± 2.34 species per reef, respectively) (Fig. 4A; Supplementary Data Fig. S1a). Furthermore, GLMM estimates indicated that reef richness was significantly affected by seasonal change (*P* < 0.0001) but it was largely insensitive to the health status of the reef (*P*-value non-significant for both status and the interaction term status × season, Table 1). This was also the case for other estimates of α-diversity such as the mean number of species per sampling quadrat (i.e. species density) and the Shannon diversity index (Fig. 4B, C;  Fig. S1b, c). Again, seasonality had a highly significant effect on both metrics (*P* < 0.0001), while the health status was non-significant (Table 1). Health status did have a small effect on modulating the seasonal pattern, as evidenced by the presence of a significant interaction. However, separate pairwise contrasts for each season showed that the only case where health status had a statistically detectable effect was for species density in summer, when the mean estimate at degraded reefs (19.4 ± 2.21 species/0.25 m^2^) was significantly larger than at healthy reefs (13.8 ± 1.63 species/0.25 m^2^) (*P *= 0.038; Table S3). In all other cases, the mean estimates recorded at healthy and degraded reefs in each season were statistically indistinguishable.

**Table 1.** Influence of kelp forest status (fixed categorical covariate with two levels: healthy and degraded) and season (fixed categorical covariate with four levels) on the α-biodiversity of seaweed assemblages found at golden kelp stands. Values are from Wald χ^2^ tests for the fixed terms fitted with a generalized linear mixed model (GLMM); σ^2^ for sampling reef as a random intercept nested in season was 0.042 for species density and 0.009 for diversity; d.f. = degrees of freedom. Significant *P*-values are shown in bold.

		Reef richness (species per reef)		Species density (species per 0.25 m^2^)		α-Diversity (Shannon, ln)	
Fixed effects	d.f.	Wald χ^2^	*P*-value	Wald χ^2^	*P*-value	Wald χ^2^	*P*-value
Kelp forest status = *St*	1	0.603	0.4374	0.264	0.607	0.147	0.701
Season = *Se*	3	52.75	**<0.0001**	130.78	**<0.0001**	95.06	**<0.0001**
*Se *× *St*	3	5.90	0.1168	27.37	**<0.0001**	11.62	**0.0088**
Model		Negative binomial (log link), dispersion modelled as a function of *St*		Poisson (log link)		Gaussian (log link), dispersion modelled as a function of *St*	

**Fig. 5.**
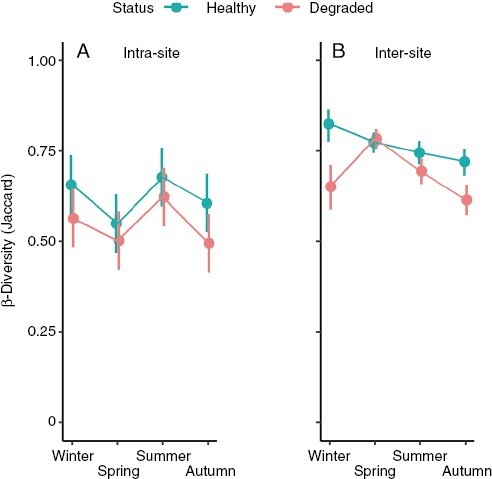
β-Biodiversity of the associated seaweed assemblage: influence of the health status (healthy vs. degraded) of golden kelp reefs on the estimates at two spatial scales: (A) intra-reef (sampling quadrats separated tens of metres) and (B) inter-reef (sampling quadrats separated hundreds to a few thousand metres). Values are least-squares means (±95 % confidence intervals) derived from GLMMs in Table 2. *N* = 40 for intra-reef means (four reefs per level of health status, 10 Jaccard distances per reef) and 50 for inter-reef means (two pairs of adjacent reefs per level of health status, 25 Jaccard distances per pair).

Unlike α-diversity, the estimates of spatial β-diversity (Jaccard) did show a moderate but significant drop with a deterioration in health status, which was perceptible at the two spatial scales analysed (Fig. 5; Supplementary Data Fig. S2). For the intra-reef scale (tens of metres), the influence of health status (*P *= 0.0094, Table 2) was consistent throughout the year (non-significant interaction term) and meant that Jaccard distances were, on average, 0.08 ± 0.029 units lower in the degraded reefs than in the healthy ones. This was equivalent to 13 % more β-diversity on healthy (0.62 ± 0.021, least-squares mean ± s.e. averaged over the four seasons) than on degraded reefs (0.55 ± 0.021). Intra-reef β-diversity also varied with the seasons (*P *= 0.0108), being higher in summer and winter while decreasing in the transition seasons when seaweed richness was changing due to the addition (spring) or loss (autumn) of summer annuals (Fig. 5A). In fact, shifts from spring to summer were significant (*P *= 0.015) ( Table S4). The magnitude of these changes (mean increase from spring to summer: 0.12 ± 0.041; mean decrease from summer to autumn: 0.10 ± 0.041) resembled the effect estimated for health status (0.08 ± 0.029), implying that kelp forest degradation had an impact on spatial β-diversity at the intra-reef scale comparable to the variation induced by seasonal change. Health status also had a significant detrimental effect on β-diversity variation at the inter-reef scale (hundreds to a few thousand kilometres; *P* < 0.0001, Table 2). Predictably, inter-reef estimates were somewhat higher than at the intra-reef scale (Fig. 5B). Nevertheless, they were again adversely affected by a deterioration in health status (*P* < 0.0001; Table 2), although in this case, the size of the effect varied with the season (*P* < 0.0001 for the interaction term). Seasonal variability was mostly due to changes in the spatial β-diversity recorded on degraded reefs. In particular, it peaked in spring, rendering mean inter-reef estimates on degraded reefs undistinguishable from those in healthy reefs (*P *= 0.615, Table S5). Nonetheless, mean β-diversity on degraded reefs remained significantly below that recorded on healthy reefs in all other seasons (Tukey adjusted *P*-values ranging from 0.0391 to <0.0001, Table S5).

**Table 2.** Influence of kelp forest status (fixed categorical covariate with two levels: healthy and degraded) and season (fixed categorical covariate with four levels) on the β-biodiversity of seaweed assemblages found at golden kelp stands. β-Biodiversity was estimated at intra-reef (pairwise Jaccard distances among sampling quadrats separated tens of metres) and inter-reef (sampling quadrats separated by hundreds to a few thousand metres) scales. Values are from Wald χ^2^ tests for the fixed terms fitted with a generalized linear mixed model (GLMM); σ^2^ for sampling reef as a random covariate nested in season was 0.005 for the intra-reef model while σ^2^ for sampling a reef pair was 0.007 for the inter-reef model; d.f. = degrees of freedom. Significant *P*-values are shown in bold.

		β-Diversity intra-reef (Jaccard)		β-Diversity inter-reef (Jaccard)	
Fixed effects	d.f.	Wald χ^2^	*P*-value	Wald χ^2^	*P*-value
Kelp forest status = *St*	1	6.746	**0.0094**	19.79	**<0.0001**
Season = *Se*	3	11.19	**0.0108**	18.50	**0.0003**
*Se *× *St*	3	0.799	0.8497	33.26	**<0.0001**
Model		Gaussian (identity link)		Beta (logit link), dispersion modelled as a function of *Se*	

## DISCUSSION

α-Diversity is commonly used in conservation and environmental impact studies, particularly in the marine environment, largely due to its simplicity and intuitive appeal (Gray, 2000; Chapman, 2022). However, obtaining reliable estimates of α-diversity can be challenging, and certain α-diversity measures have demonstrated limited discriminatory power (Morris *et al.*, 2014; Firth *et al.*, 2016; Chapman, 2022). The latter appears to be the case in our study. The assemblages of our healthy and degraded reefs were noticeably distinct, as indicated by the vegetation layer results. Nevertheless, none of our measures of α-diversity detected significant differences between healthy and degraded reefs. In fact, the mean α-diversity values calculated for degraded reefs were occasionally nearly indistinguishable from those associated with healthy reefs. This suggests that while the loss of the kelp canopy may result in a community with a different composition and appearance, it may not necessarily lead to lower richness.

The lack of sensitivity in detecting changes in community composition with a metric such as α-diversity, which ignores species identity, is unsurprising. Interestingly, our results also suggest that another equally simple metric, which similarly ignores species identity, is capable of discriminating degraded reefs using the same raw information. Thus, our β-diversity estimates revealed significantly lower values in degraded reefs compared to healthy ones. β-Diversity estimates do not replace α-diversity metrics but complement them, since β-diversity assesses other community attributes, such as spatial variation. This possibly explains why β-diversity may be particularly sensitive to perturbations that alter the physical appearance of the community. Our findings indicate that the removal of kelp canopy results in assemblages with greater spatial homogeneity, a finding that is consistent with the homogenization reported in other cases where kelp forests were replaced by a turf that still retained substantial species richness, but with a simpler physical structure (Filbee‐Dexter and Wernberg, 2018; Pessarrodona *et al.*, 2021). Furthermore, our results also indicate that the homogenization extends beyond the local scale since the loss of kelp canopy also lowered the heterogeneity between reefs situated a few kilometres apart. This suggests that the macroalgal assemblages are somewhat more similar across degraded reefs than across healthy ones.

Other studies of marine benthic assemblages have found that β-diversity may have a positive correlation with environmental heterogeneity (Anderson *et al.*, 2006). This could potentially explain the observed decrease in spatial variation found in our degraded reefs. Like other foundation species, kelps modify physical conditions (Altieri and Koppel, 2014). In our healthy reefs, the kelp canopy is distributed in a patchy manner, resulting in areas with varying densities of kelp with predictable consequences for factors such as water movement and light availability. Therefore, it seems reasonable to anticipate that the loss of the kelp canopy possibly leads to a more homogeneous physical environment. This, in turn, could favour the development of an assemblage with an equally more uniform spatial distribution.

Our results support the idea that β-diversity is more appropriate than α-diversity for detecting the assemblage changes caused by kelp canopy loss. Moreover, the effect of canopy loss on β-diversity was insensitive to seasonal change, especially at the local scale. This behaviour contrasts with the seasonal variability of α-diversity and suggests that β-diversity may also be a more robust approach. It is worth noting that the greater robustness and sensitivity of β-diversity does not imply a larger sampling effort, as both metrics are based on the same raw data. Moreover, the fact that our measures of β-diversity are based on an index that uses only presence/absence data suggests that the effort of field data collection work could be reduced without necessarily losing sensitivity.

Other studies have also reported cases where β-diversity variation showed significant responses to perturbation, even when no effects on the structure and/or α-diversity were apparent (Bevilacqua *et al.*, 2012). However, there are also instances where both α- and β-diversity were equally affected (O’Shaughnessy *et al.*, 2023). Presumably, this difference may be due to the type of perturbance, as β-diversity may be particularly sensitive to disturbances, either natural or human-induced, that alter the spatial heterogeneity of the assemblage rather than its composition. Nevertheless, exploring changes in β-diversity variation may still be useful, even when the perturbation affects the assemblage structure and/or α-diversity (Bevilacqua *et al.*, 2012). Furthermore, a better assessment of the impact of human activities could be achieved by combining multiple metrics and spatial scales (O’Shaughnessy *et al.*, 2023).

In contrast to manipulative experiments, observational experiments such as this are by definition restricted to locations where natural occurrences of the phenomenon of interest can be observed. While this approach offers increased realism, it also poses a risk of confounding variables that may influence the outcome. In our case, a more robust demonstration of the impact of kelp canopy collapse on diversity patterns would ideally be achieved if healthy and overgrazed reefs were spatially intermixed with each other. Unfortunately, our overgrazed reefs were located on the inner side of the islands found at the entrance of the estuary, while healthy reefs were situated on the mainland coast near those islands. As a result, it cannot be definitively disregarded that differences in wave exposure did not contribute to the diversity patterns we observed. It is common to encounter non-ideal spatial arrangements of sampling sites in observational fieldwork (Butsic *et al.*, 2017), but obtaining information on potential confounding variables can aid in assessing their potential influence. Our environmental evidence (fetch, significant wave height, water temperature, depth, bottom sediment texture, dissolved nutrient concentration) did not indicate consistent differences between the healthy and degraded groups of reefs (Barrientos *et al.*, 2022*a*). This supports the conclusion that kelp forest degradation caused by overgrazing is the actual cause of the observed differences in diversity patterns. Nevertheless, further studies with different sampling designs and in other regions seem warranted to corroborate the commonality of this effect.

Our findings provide novel insights into the ecological significance of kelp forests in shaping the dynamics of seaweed communities in NW Iberia, a previously overlooked aspect. Our results revealed distinct patterns in species composition between degraded and healthy kelp forests. In degraded reefs, the coralline algae *Lithophyllum hibernicum* and *Corallina officinalis* consistently dominated throughout the year, with a notable increase of turf species during spring and summer, especially the invasive *Asparagopsis armata* stadium *Falkenbergia rufolanosa*. In contrast, healthy kelp forests exhibited a relatively stable community structure throughout the year, with only a slight increase in turf and subcanopy species during the spring, mostly *Acrosorium ciliolatum* and *Dictyota dichotoma*, respectively.

At healthy reefs, the observed community composition is consistent with the typical stratified configuration of kelp forests, where kelp forms an upper canopy above an erect and prostrate understorey. The lower layers consist of algal turf assemblages composed of species, including branched and encrusting corallines together with fleshy foliose and filamentous red algae (Dayton, 1985). The seasonal dynamics observed in our study, evident in both healthy and degraded reefs, are consistent with subtidal seaweed communities where seasonal and ephemeral species emerge during warmer seasons and decrease during autumn and winter, leaving behind perennial and pseudo-perennial species (Reed and Foster, 1984; Santelices and Ojeda, 1984; Johnson and Mann, 1988; 1993). Despite the increase of turf species also at healthy reefs in summer, our results demonstrate the regulatory role of kelp forests in preventing the overgrowth of seasonal and ephemeral turf species, particularly invasive ones such as *A. armata* stadium *F. rufolanosa*, which was the most abundant turf species in our study area’s degraded reefs during summer. This can be attributed to the shading and physical effect of kelps (Reed and Foster, 1984), and highlights the role of canopy-forming species in maintaining relatively pristine areas by restricting the growth of turf-forming and invasive algae (Benedetti-Cecchi *et al.*, 2001).

Our findings did not reveal significant changes between healthy and degraded reefs for any of the α-diversity metrics (richness, species density, Shannon index). This can be attributed to the proliferation of algal turf in the degraded reefs. Although turfs are commonly perceived as having low diversity and being largely monospecific, recent studies employing molecular tools have demonstrated that they harbour more diversity than meets the eye (Piñeiro-Corbeira *et al.*, 2019, 2020; Díaz-Tapia *et al.*, 2021). This fact also accounts for the homogenization observed in our degraded reefs, where the decline in β-diversity compared to healthy reefs was evident at both intra- and inter-reef scales. These results align with global patterns observed in temperate reefs across the world, where kelp forests have dwindled in recent decades, accompanied by an increase in algal turfs (Pessarrodona *et al.*, 2021). Such findings are not surprising, as biodiversity patterns are strongly influenced by the interactions between the canopy and understorey, and the heterogeneity of the habitat (Smale *et al.*, 2011). Disturbances can significantly alter these patterns, leading to changes in β-diversity (Piazzi and Ceccherelli, 2020). For example, sediment disturbances (Balata *et al.*, 2007) and invasions (Piazzi and Balata, 2008) have been linked to the loss of β-diversity at small and large spatial scales, as they promote the colonization of turf and/or opportunistic species, ultimately homogenizing the habitat. Many areas experiencing kelp forest degradation have reported an increase in turf abundance accompanied by elevated sedimentation, as numerous turf species possess the ability to retain substantial sediment loads and modify ecosystem dynamics (Pessarrodona *et al.*, 2021).

To fully understand and identify the processes underlying the loss of marine habitats such as kelp forests, knowledge of the biodiversity patterns across both temporal and spatial scales is crucial (Piazzi and Ceccherelli, 2020; Catalan *et al.*, 2023). Surprisingly, β-diversity has been largely overlooked in the context of understanding ecosystem functioning under pressure, despite its potential to detect changes in community structure at different scales (Piazzi and Ceccherelli, 2020). Our results suggest that relying solely on patterns of richness and other α-diversity metrics can potentially miss some of the consequences of kelp forest loss on the organization of the associated community.

## CONCLUSIONS

This study represents an initial step toward understanding how β-diversity measures can elucidate the effects of disturbances on kelp forests. Our findings underline the importance of integrating small-scale β-diversity when evaluating the repercussions of kelp deforestation on the associated biotic community. Sole reliance on species richness and α-diversity may inadvertently neglect changes in the spatial dimension of biotic diversity. Therefore, incorporating β-diversity can enhance the development of management and conservation strategies for kelp forests. Nevertheless, the sensitivity of β-diversity estimates to natural variability necessitates a careful sampling/study design to minimize potential confounding factors. Our results emphasize the significance of adopting a comprehensive approach that considers both α- and β-diversity, as well as spatial and temporal scales, when assessing the impact of disturbances on marine ecosystems, such as kelp forests. However, to ascertain the broader applicability of our observed patterns in response to kelp deforestation, further assessments of both α- and β-diversity in other temperate zones seem warranted. These additional studies will contribute to a more comprehensive understanding of the ecological consequences of kelp canopy loss and provide valuable insights for effective conservation and management efforts.

## SUPPLEMENTARY DATA

Supplementary data are available at *Annals of Botany* online and consist of the following.

Table S1: Reef pairs used for β-biodiversity estimates at the inter-reef scale. Table S2. List of the species found in our study including the vegetation layer categories. Table S3: Results of post-hoc tests for least-square mean estimates of species density (species/0.25 m^2^). Table S4: Results of post-hoc tests for least-square mean estimates of β-diversity at the intra-reef scale (tens of metres). Table S5: Results of post-hoc tests for least-square mean estimates of β-diversity at the inter-reef scale (hundreds to a few thousand metres). Fig. S1: α-Biodiversity of the associated seaweed assemblage: influence of health status (healthy vs. degraded) of golden kelp reefs on (a) total number of seaweed species per reef, (b) species density and (c) Shannon diversity. Fig. S2: β-Biodiversity of the associated seaweed assemblage: influence of health status (healthy vs. degraded) of ngolden kelp reefs on the estimates at two spatial scales. SI1: Wind fetch for sampling sites.

mcad154_suppl_Supplementary_Data

mcad154_suppl_Supplementary_Figures_S1

mcad154_suppl_Supplementary_Figures_S2

mcad154_suppl_Supplementary_Tables_S1

mcad154_suppl_Supplementary_Tables_S2

mcad154_suppl_Supplementary_Tables_S3

mcad154_suppl_Supplementary_Tables_S4

mcad154_suppl_Supplementary_Tables_S5
