# Supplementary material for: Kelp forests collapse reduces understorey seaweed β-diversity
Source: Ann Bot. 2023 Oct 10;133(1):93–104. doi: 10.1093/aob/mcad154 (PMC10921829; doi:10.1093/aob/mcad154)
Supplement: mcad154_suppl_Supplementary_Data [file mcad154_suppl_supplementary_data.docx]

# Wind and wave exposure assessment at sampling sites

Fetch (i.e. the unobstructed length of water over which wind from a certain direction can blow over) was calculated to assess wind and wave exposure. At each site, we measured the length of 36 equiangular fetch vectors (i.e. one fetch vector per 10 degrees), with maximum distance set at 300 km. To ascertain the overall and quadrant-specific exposure at each site, we computed the mean length of the 36 fetch vectors and of the 9 fetch vectors within each quadrant, respectively. Fetch calculation and visualization was accomplished using the *fetchR* package (Seers, 2018, Posit team, 2023, R Core Team, 2023).

The degraded sites exhibited the most disparate results in terms of overall fetch, with site S7 recording the lowest value (5.5 km) and site S3 registering the largest (72.4 km). Meanwhile, healthy reefs showed intermediate values, falling within the range observed in the degraded sites (Table 1). The contribution of the north and east quadrants to the overall fetch was low across all sites. Instead, two healthy (S5 and S6) and one degraded (S4) sites were predominantly exposed to the west, while two healthy (S1 and S2) and one degraded (S3) reefs displayed considerable exposure towards the south (Fig. 1).

Table 1. Wind and wave exposure. Overall and quadrant-specific fetch estimates (km). Values are averages for 36 (overall) and 9 (quadrant-specific) fetch vectors. Maximum distance set at 300 km.

| Reef status | Site | Fetch (km) | | | | |
| --- | --- | --- | --- | --- | --- | --- |
|  |  | Overall | North | East | South | West |
| Healthy | S1 | 19.3 | 0.1 | 0.7 | 71.9 | 4.6 |
|  | S2 | 27.2 | 0.2 | 0.1 | 72.6 | 35.9 |
|  | S5 | 28.6 | 7 | 1.4 | 1 | 104.8 |
|  | S6 | 36.9 | 4.5 | 2.6 | 35 | 105.6 |
| Degraded | S3 | 72.4 | 9.2 | 9.6 | 137.3 | 133.4 |
|  | S4 | 29.1 | 2.3 | 9.9 | 3.7 | 100.4 |
|  | S7 | 5.5 | 10 | 8.2 | 3.7 | 0.1 |
|  | S8 | 6.7 | 12.1 | 9 | 5.4 | 0.4 |


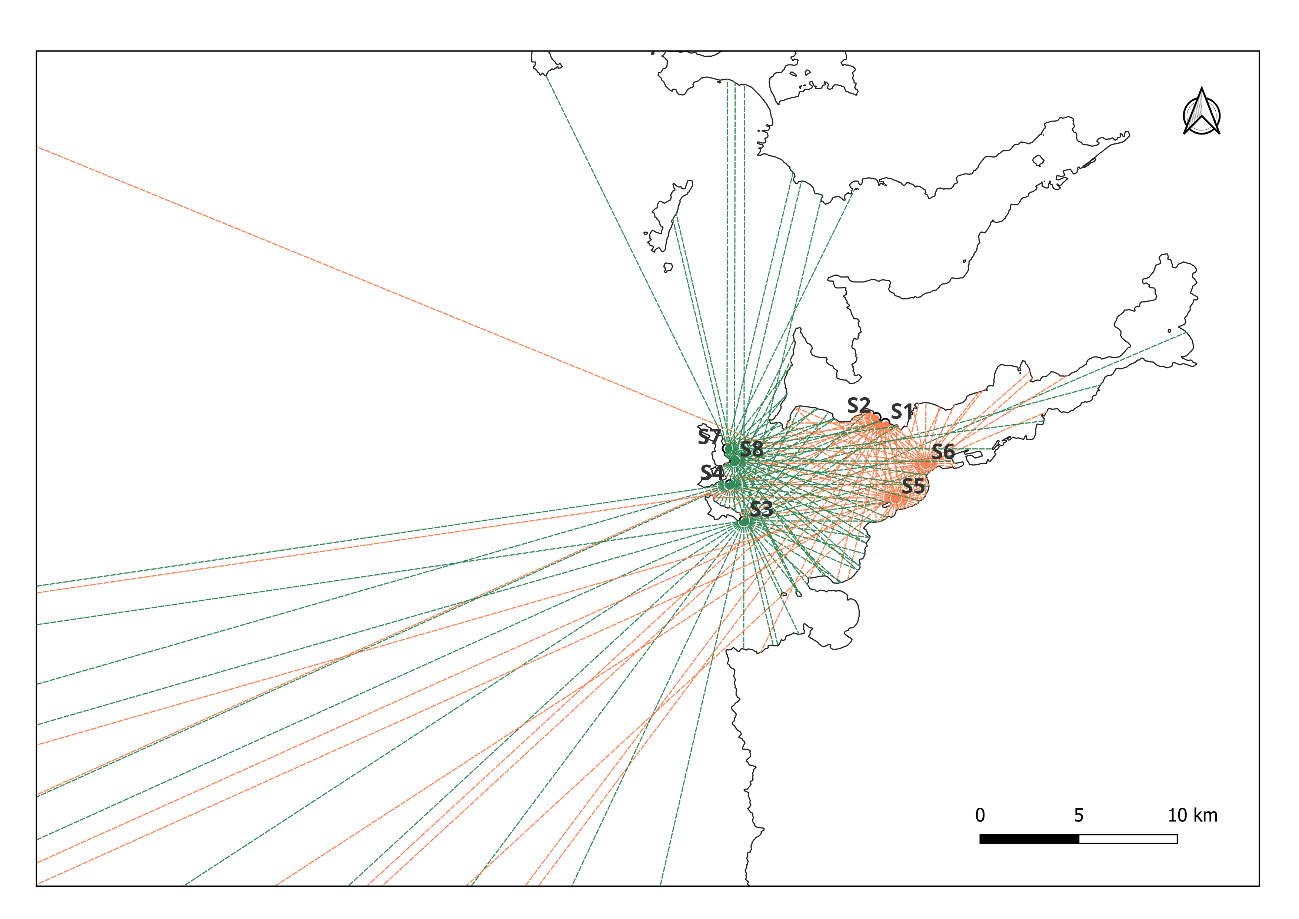


Fig. 1. Map displaying wave and wind exposure patterns at the sampled sites. Fetch vectors calculated for healthy (orange) and degraded (green) reefs.

## References

**Posit team.** **2023.** RStudio: Integrated Development Environment for R. Boston, MA: Posit Software, PBC.

**R Core Team.** **2023.** R: A Language and Environment for Statistical Computing. Vienna, Austria: R Foundation for Statistical Computing.

**Seers B.** **2018.** fetchR: Calculate Wind Fetch. R package version 2.1-1.
