# Supplementary material for: Kelp forests collapse reduces understorey seaweed β-diversity
Source: Ann Bot. 2023 Oct 10;133(1):93–104. doi: 10.1093/aob/mcad154 (PMC10921829; doi:10.1093/aob/mcad154)
Supplement: mcad154_suppl_Supplementary_Figures_S1 [file mcad154_suppl_supplementary_figures_s1.docx]

Supplementary Information

##
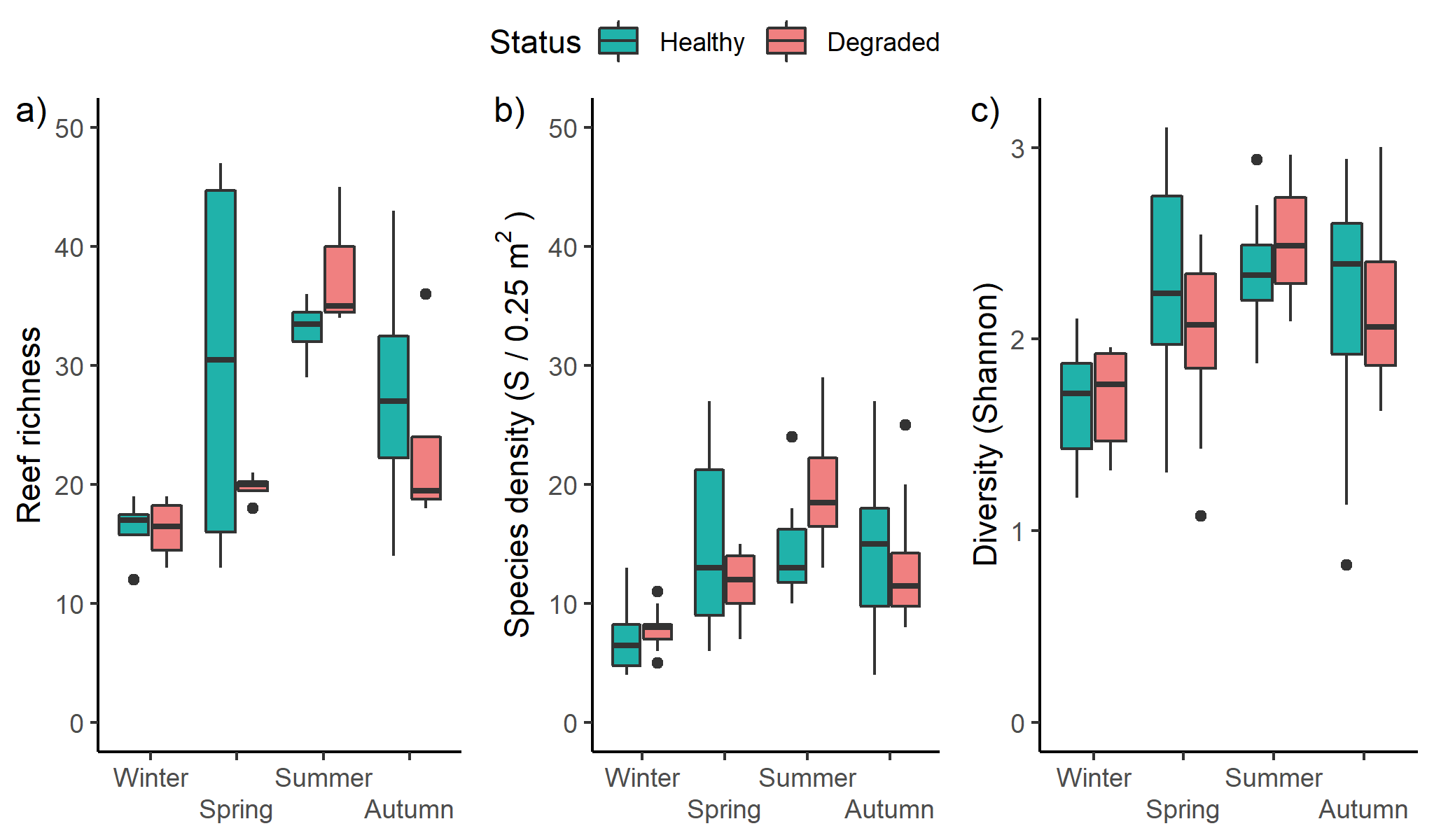


Fig. S1. α-biodiversity of the associated seaweed assemblage: influence of the conservation status (healthy vs. degraded) of golden kelp reefs on (a) total number of seaweed species per reef, (b) species density, and (c) Shannon diversity. From bottom to top, each box-plot shows the minimum, first quartile, median, third quartile and maximum. Values distant from the edges of the box more than 1.5 times the interquartile range shown as dots. N = 4 for box-plots in (a) (four sites per level of conservation status) and 20 for box-plots in (b) and (c) (four sites per level of conservation status, five sampling quadrats per site).
