# Supplementary material for: Kelp forests collapse reduces understorey seaweed β-diversity
Source: Ann Bot. 2023 Oct 10;133(1):93–104. doi: 10.1093/aob/mcad154 (PMC10921829; doi:10.1093/aob/mcad154)
Supplement: mcad154_suppl_Supplementary_Figures_S2 [file mcad154_suppl_supplementary_figures_s2.docx]

Supplementary Information


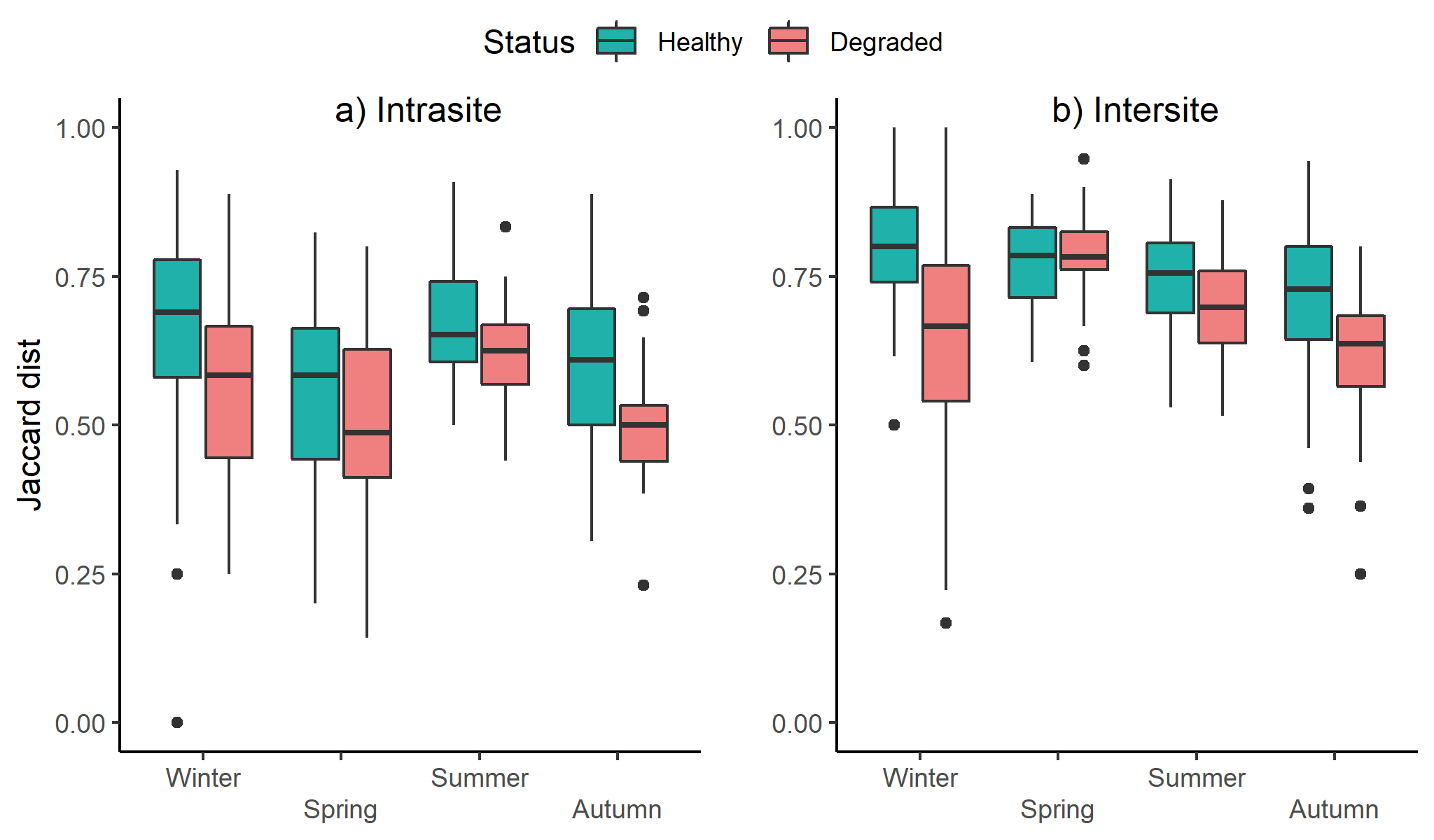


Fig. S2. β-biodiversity of the associated seaweed assemblage: influence of the conservation status (healthy vs. degraded) of golden kelp reefs on the estimates at two spatial scales: (a) intrasite (sampling quadrats separated tens of meters) and (b) intersite (sampling quadrats separated hundreds to a few thousand meters). From bottom to top, each box-plot shows the minimum, first quartile, median, third quartile and maximum. Values distant from the edges of the box more than 1.5 times the interquartile range shown as dots. N = 40 for intrasite box-plots (four sites per level of conservation status, 10 Jaccard distances per site) and 50 for intersite ones (two pairs of adjacent sites per level of conservation status, 25 Jaccard distances per pair).
