# Supplementary material for: Kelp forests collapse reduces understorey seaweed β-diversity
Source: Ann Bot. 2023 Oct 10;133(1):93–104. doi: 10.1093/aob/mcad154 (PMC10921829; doi:10.1093/aob/mcad154)
Supplement: mcad154_suppl_Supplementary_Tables_S1 [file mcad154_suppl_supplementary_tables_s1.docx]

Supplementary Information

Table. S1. Site pairs used for β-biodiversity estimates at intersite scale.

| Pair | Status | Sites | Distance by sea (m) |
| --- | --- | --- | --- |
| A | Healthy | S1-S2 | 750 |
| B | Degraded | S3-S4 | 2580 |
| C | Healthy | S5-S6 | 2800 |
| D | Degraded | S7-S8 | 950 |
