# Supplementary material for: Kelp forests collapse reduces understorey seaweed β-diversity
Source: Ann Bot. 2023 Oct 10;133(1):93–104. doi: 10.1093/aob/mcad154 (PMC10921829; doi:10.1093/aob/mcad154)
Supplement: mcad154_suppl_Supplementary_Tables_S2 [file mcad154_suppl_supplementary_tables_s2.docx]

Supplementary Information

Table S2: List of the species found in our study including the vegetation layer categories

| **Species** | **Vegetation layer** | **Phylum** |
| --- | --- | --- |
| *Bryopsis plumosa* | Turf | Chlorophyta |
| *Cladophora hutchinsiae* | Turf | Chlorophyta |
| *Cladophora lehmanniana* | Turf | Chlorophyta |
| *Codium fragile* | Canopy | Chlorophyta |
| *Codium tomentosum* | Canopy | Chlorophyta |
| *Codium vermilara* | Canopy | Chlorophyta |
| *Derbesia marina* | Turf | Chlorophyta |
| *Ulva clathrata* | Turf | Chlorophyta |
| *Ulva rigida* | Subcanopy | Chlorophyta |
| *Cutleria multifida* stadium *Aglaozonia parvula* | Crust | Ochrophyta |
| *Carpomitra costata* | Subcanopy | Ochrophyta |
| *Cladostephus spongiosus* | Subcanopy | Ochrophyta |
| *Colpomenia peregrina* | Subcanopy | Ochrophyta |
| *Desmarestia ligulata* | Canopy | Ochrophyta |
| *Dictyopteris lucida* | Canopy | Ochrophyta |
| *Dictyopteris polypodioides* | Canopy | Ochrophyta |
| *Dictyota dichotoma* | Subcanopy | Ochrophyta |
| *Halidrys siliquosa* | Canopy | Ochrophyta |
| *Halopteris filicina* | Turf | Ochrophyta |
| *Halopteris scoparia* | Subcanopy | Ochrophyta |
| *Laminaria ochroleuca* | Canopy | Ochrophyta |
| *Padina pavonica* | Subcanopy | Ochrophyta |
| *Saccorhiza polyschides* | Canopy | Ochrophyta |
| *Sargassum muticum* | Canopy | Ochrophyta |
| *Sphacelaria plumula* | Turf | Ochrophyta |
| *Taonia atomaria* | Subcanopy | Ochrophyta |
| *Gongolaria baccata* | Canopy | Ochrophyta |
| *Gongolaria usneoides* | Canopy | Ochrophyta |
| *Undaria pinnatifida* | Canopy | Ochrophyta |
| *Zanardinia typus* | Crust | Ochrophyta |
| *Acrosorium ciliolatum* | Turf | Rhodophyta |
| *Aglaothamnion pseudobyssoides* | Turf | Rhodophyta |
| *Aglaothamnion tenuissimum* | Turf | Rhodophyta |
| *Aglaothamnion tripinnatum* | Turf | Rhodophyta |
| *Ahnfeltia plicata* | Subcanopy | Rhodophyta |
| *Ahnfeltiopsis devoniensis* | Subcanopy | Rhodophyta |
| *Anotrichium furcellatum* | Turf | Rhodophyta |
| *Antithamnionella spirographidis* | Turf | Rhodophyta |
| *Antithamnionella ternifolia* | Turf | Rhodophyta |
| *Apoglossum ruscifolium* | Turf | Rhodophyta |
| *Asparagopsis armata* | Subcanopy | Rhodophyta |
| *Asparagopsis armata* stadium *Falkenbergia rufolanosa* | Turf | Rhodophyta |
| *Bonnemaisonia asparagoides* | Subcanopy | Rhodophyta |
| *Bonnemaisonia hamifera* stadium *Trailiella intricata (stadium)* | Turf | Rhodophyta |
| *Calliblepharis ciliata* | Subcanopy | Rhodophyta |
| *Calliblepharis jubata* | Subcanopy | Rhodophyta |
| *Callithamnion tetragonum* | Turf | Rhodophyta |
| *Ceramium ciliatum* | Turf | Rhodophyta |
| *Ceramium cimbricum* | Turf | Rhodophyta |
| *Ceramium diaphanum* | Turf | Rhodophyta |
| *Ceramium echionotum* | Turf | Rhodophyta |
| *Ceramium pallidum* | Turf | Rhodophyta |
| *Ceramium secundatum* | Turf | Rhodophyta |
| *Champia parvula* | Turf | Rhodophyta |
| *Chondracanthus acicularis* | Subcanopy | Rhodophyta |
| *Chondracanthus teedei* | Subcanopy | Rhodophyta |
| *Chondria coerulescens* | Turf | Rhodophyta |
| *Chondrus crispus* | Subcanopy | Rhodophyta |
| *Compsothamnion thuioides* | Turf | Rhodophyta |
| *Corallina officinalis* | Turf | Rhodophyta |
| *Crouania attenuata* | Turf | Rhodophyta |
| *Cryptonemia lomation* | Subcanopy | Rhodophyta |
| *Cryptopleura ramosa* | Turf | Rhodophyta |
| *Dasya hutchinsiae* | Turf | Rhodophyta |
| *Dasya sessilis* | Turf | Rhodophyta |
| *Dasysiphonia japonica* | Turf | Rhodophyta |
| *Dilsea carnosa* | Subcanopy | Rhodophyta |
| *Erythroglossum laciniatum* | Turf | Rhodophyta |
| *Erythroglossum lusitanicum* | Turf | Rhodophyta |
| *Gaillona hookeri* | Turf | Rhodophyta |
| *Gastroclonium ovatum* | Subcanopy | Rhodophyta |
| *Gayliella flaccida* | Turf | Rhodophyta |
| *Gelidium corneum* | Subcanopy | Rhodophyta |
| *Gelidium spinosum* | Subcanopy | Rhodophyta |
| *Gloiocladia repens* | Subcanopy | Rhodophyta |
| *Gracilaria multipartita* | Subcanopy | Rhodophyta |
| *Grateloupia turuturu* | Subcanopy | Rhodophyta |
| *Gymnogongrus crenulatus* | Subcanopy | Rhodophyta |
| *Halurus equisetifolius* | Turf | Rhodophyta |
| *Halurus flosculosus* | Turf | Rhodophyta |
| *Herposiphonia secunda* | Turf | Rhodophyta |
| *Herposiphonia secunda f. tenella* | Turf | Rhodophyta |
| *Heterosiphonia plumosa* | Subcanopy | Rhodophyta |
| *Hypoglossum hypoglossoides* | Turf | Rhodophyta |
| *Jania longifurca* | Turf | Rhodophyta |
| *Jania rubens* | Turf | Rhodophyta |
| *Jania squamata* | Turf | Rhodophyta |
| *Kallymenia reniformis* | Subcanopy | Rhodophyta |
| *Lithophyllum hibernicum* | Crust | Rhodophyta |
| *Lomentaria clavellosa* | Turf | Rhodophyta |
| *Lomentaria orcadensis* | Turf | Rhodophyta |
| *Melanothamnus harveyi* | Turf | Rhodophyta |
| *Mesophyllum expansum* | Crust | Rhodophyta |
| *Mesophyllum lichenoides* | Crust | Rhodophyta |
| *Metacallophyllis laciniata* | Subcanopy | Rhodophyta |
| *Monosporus pedicellatus* | Turf | Rhodophyta |
| *Neurocaulon foliosum* | Subcanopy | Rhodophyta |
| *Nitophyllum punctatum* | Subcanopy | Rhodophyta |
| *Osmundea pinnatifida* | Turf | Rhodophyta |
| *Peyssonnelia atropurpurea* | Crust | Rhodophyta |
| *Phyllophora crispa* | Subcanopy | Rhodophyta |
| *Phyllophora sicula* | Subcanopy | Rhodophyta |
| *Plocamium cartilagineum* | Subcanopy | Rhodophyta |
| *Plocamium raphelisianum* | Subcanopy | Rhodophyta |
| *Polysiphonia delicata* | Turf | Rhodophyta |
| *Polysiphonia denudata* | Turf | Rhodophyta |
| *Pterocladiella capillacea* | Subcanopy | Rhodophyta |
| *Pterosiphonia complanata* | Turf | Rhodophyta |
| *Pterothamnion crispum* | Turf | Rhodophyta |
| *Rhodophyllis divaricata* | Subcanopy | Rhodophyta |
| *Rhodothamniella floridula* | Turf | Rhodophyta |
| *Rhodymenia holmesii* | Turf | Rhodophyta |
| *Rhodymenia pseudopalmata* | Turf | Rhodophyta |
| *Schottera nicaeensis* | Turf | Rhodophyta |
| *Scinaia interrupta* | Subcanopy | Rhodophyta |
| *Sphaerococcus coronopifolius* | Subcanopy | Rhodophyta |
| *Sphondylothamnion multifidum* | Turf | Rhodophyta |
| *Symphyocladiella arecina* | Turf | Rhodophyta |
| *Vertebrata fruticulosa* | Turf | Rhodophyta |
| *Vertebrata fucoides* | Turf | Rhodophyta |
| *Vertebrata furcellata* | Turf | Rhodophyta |
| *Xiphosiphonia ardreana* | Turf | Rhodophyta |
| *Xiphosiphonia pennata* | Turf | Rhodophyta |
