# Supplementary material for: Kelp forests collapse reduces understorey seaweed β-diversity
Source: Ann Bot. 2023 Oct 10;133(1):93–104. doi: 10.1093/aob/mcad154 (PMC10921829; doi:10.1093/aob/mcad154)
Supplement: mcad154_suppl_Supplementary_Tables_S3 [file mcad154_suppl_supplementary_tables_s3.docx]

Supplementary Information

Table S3. Results of post-hoc test for least-square mean estimates of Species density
(species / 0.25 m^2^). Separate pairwise tests for the influence of conservation status were conducted for each season as the interaction term was significant (see Table 1 in main text). P-values adjusted by the Tukey HSD method. Significant p-values highlighted in bold.

| Contrast: Healthy – Degraded reef | | |
| --- | --- | --- |
| Season | t ratio | p-value |
| Winter | -0.514 | 0.6079 |
| Spring | 1.456 | 0.1475 |
| Summer | -2.095 | **0.0379** |
| Autumn | 0.346 | 0.7301 |
