# Supplementary material for: Kelp forests collapse reduces understorey seaweed β-diversity
Source: Ann Bot. 2023 Oct 10;133(1):93–104. doi: 10.1093/aob/mcad154 (PMC10921829; doi:10.1093/aob/mcad154)
Supplement: mcad154_suppl_Supplementary_Tables_S4 [file mcad154_suppl_supplementary_tables_s4.docx]

Supplementary Information

Table S4. Results of post-hoc test for least-square mean estimates of β-diversity at intrasite scale (tens of meters). P-values for factor *Season* adjusted by the Tukey HSD method. Results for each *Season* averaged over the levels of *Conservation Status* since the interaction term was non-significant (see Table 2 in main text). Significant or marginally significant p-values highlighted in bold and underlined, respectively

| Contrast | t ratio | p-value |
| --- | --- | --- |
| Winter - Spring | 2.050 | 0.1721 |
| Winter - Summer | -0.951 | 0.7771 |
| Winter - Autumn | 1.454 | 0.4667 |
| Spring - Summer | -3.001 | **0.0153** |
| Spring - Autumn | -0.596 | 0.9332 |
| Summer - Autumn | 2.405 | 0.0782 |
